# Supplementary material for: Paternal DDT exposure induces sex-specific programming of fetal growth, placenta development and offspring’s health phenotypes in a mouse model
Source: Sci Rep. 2024 Mar 30;14:7567. doi: 10.1038/s41598-024-58176-7 (PMC10981700; doi:10.1038/s41598-024-58176-7)
Supplement: Supplementary file 1 — Supplementary Tables. [file 41598_2024_58176_MOESM1_ESM.docx]

**Supplementary Tables**

**Table S1**-Paternal DDT study: Number of litters and fathers represented

in each experimental endpoint

| **Endpoint** | **Group** | **Number of litters/fathers** |
| --- | --- | --- |
| Birthweight/litter size/ sex distribution |  |  |
|  | CO | 10 litters/8 fathers |
|  | DDT | 9 litters/7 fathers |
| Placenta endpoints |  |  |
|  | CO | 4 litters/4 fathers |
|  | DDT | 3 litters/3 fathers |
| Glycogen levels |  |  |
|  | CO | 4 litters/4 fathers |
|  | DDT | 3 litters/3 fathers |
| Western-blots |  |  |
|  | CO | 4 litters/4 fathers |
|  | DDT | 3 litters/3 fathers |
| Metabolic function |  |  |
|  | CO | 6 litters/6 fathers |
|  | DDT | 5 litters/4 fathers |

| **Company** | **Catalog #** | **Antibody** | **Application/Dilution** |
| --- | --- | --- | --- |
| Abcam | ab177941 | Anti-OGT / O-Linked N-Acetylglucosamine Transferase Monoclonal Antibody (EPR12713) | Wester-blot  1:1000 |
| Cell Signaling Technology | 24083 | Anti-OGT/ O-Linked N-Acetylglucosamine Transferase Monoclonal Antibody(D1D8Q) | IHC  1:200 |
| Santa Cruz Biotechnology | SC-59623 | Anti-O-GlcNAc Monoclonal Antibody (CTD110.6) | Wester-blot  1:1000 |
| Cell Signaling Technology | 5246 | Anti-EZH2 Monoclonal Antibody (D2C9) | Wester-blot  1:1000 |
| ProteinTech | 66037 | Anti-ATP5A1 Monoclonal Antibody | Wester-blot  1:5000 |
| Abcam | ab8229 | Anti-Beta Actin Polyclonal Antibody | Wester-blot  1:1000 |

**Table S2**-List of Antibodies Used for Western-Blots or Immmohistochemistry (IHC)
