# Supplementary material for: Paternal DDT exposure induces sex-specific programming of fetal growth, placenta development and offspring’s health phenotypes in a mouse model
Source: Sci Rep. 2024 Mar 30;14:7567. doi: 10.1038/s41598-024-58176-7 (PMC10981700; doi:10.1038/s41598-024-58176-7)
Supplement: Supplementary file 2 — Supplementary Figures. [file 41598_2024_58176_MOESM2_ESM.pdf]

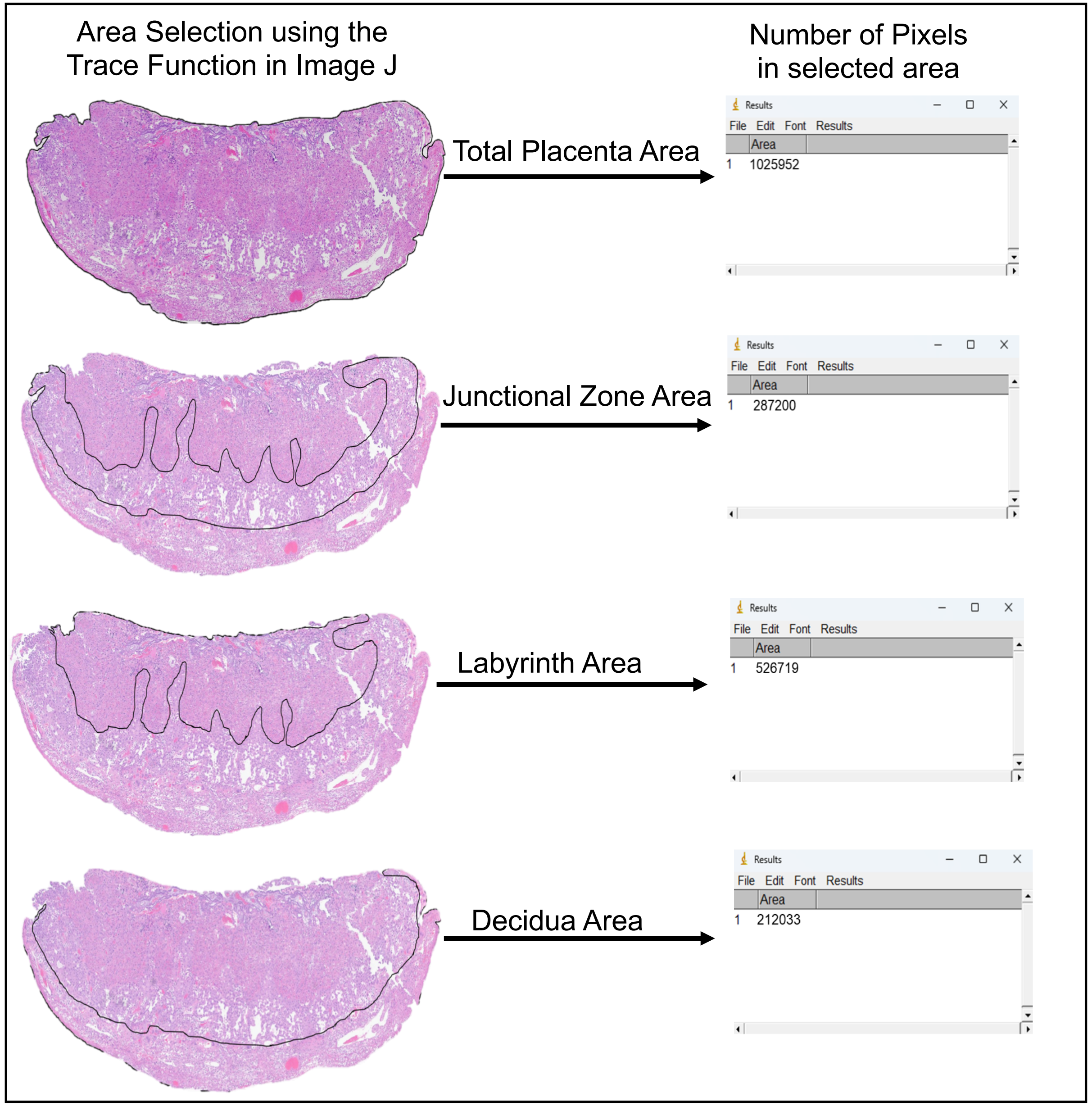

Fig. S1a. Method used to estimate total or layer-specific placenta surface area: An outline of each of two consecutive mid-sagittal placenta section images was traced, using the Image J software. The number of pixels in the traced area (black lines) was then calculated by the software. These procedures were then repeated to determine the area of each placenta layer.

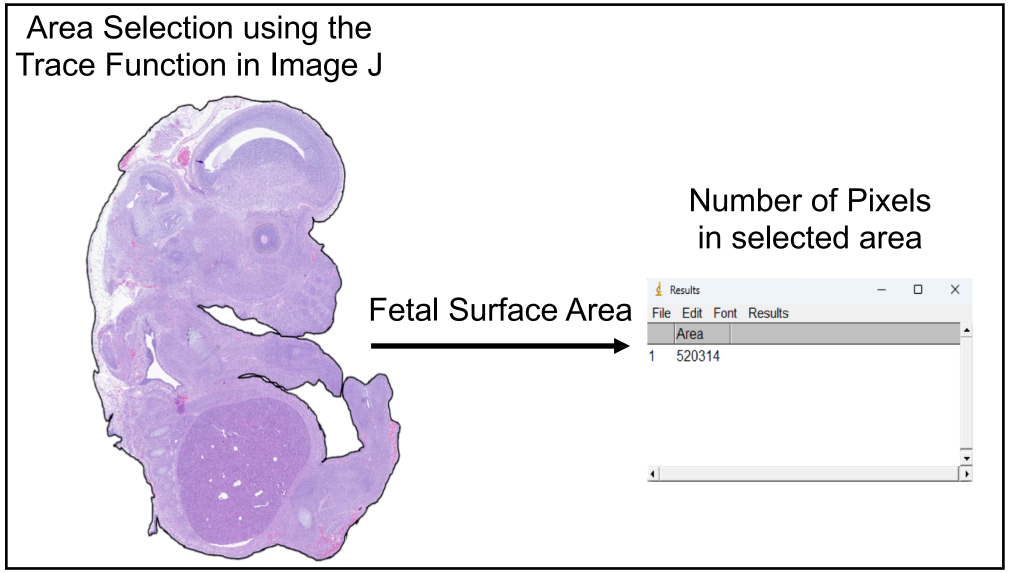

Fig. S1b. Method used to estimate fetal surface area: An outline of each of two consecutive mid-sagittal fetal section images was traced, using the Image J software. The number of pixels in the traced area (black lines) was then calculated by the software.

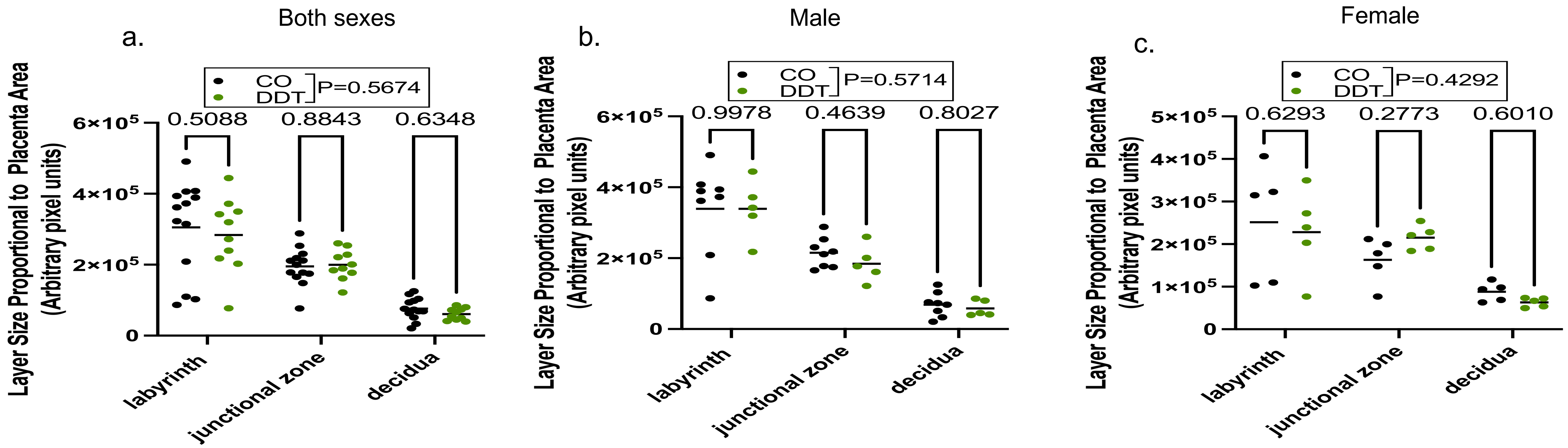

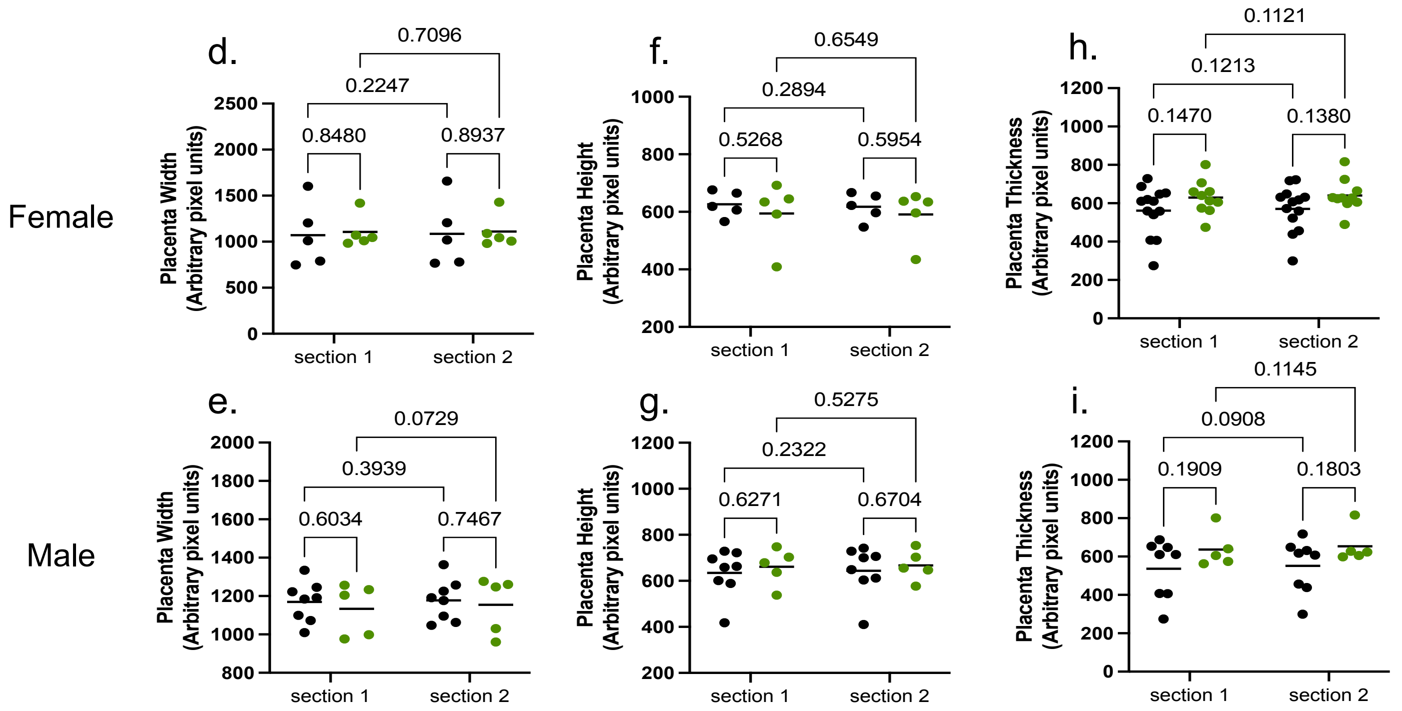

Fig. S2. (a-c) Layer-specific placenta surface area (absolute values) shown by group (a) or by group and sex (b,c). (d-i) Placenta width (d-e), height (f-g) and thickness (h-i). Data were analyzed by two-way ANOVA (a-c) or repeated measures two-way ANOVA (d-i); n=10-13/group (a-b), n=5-8 (b-i).

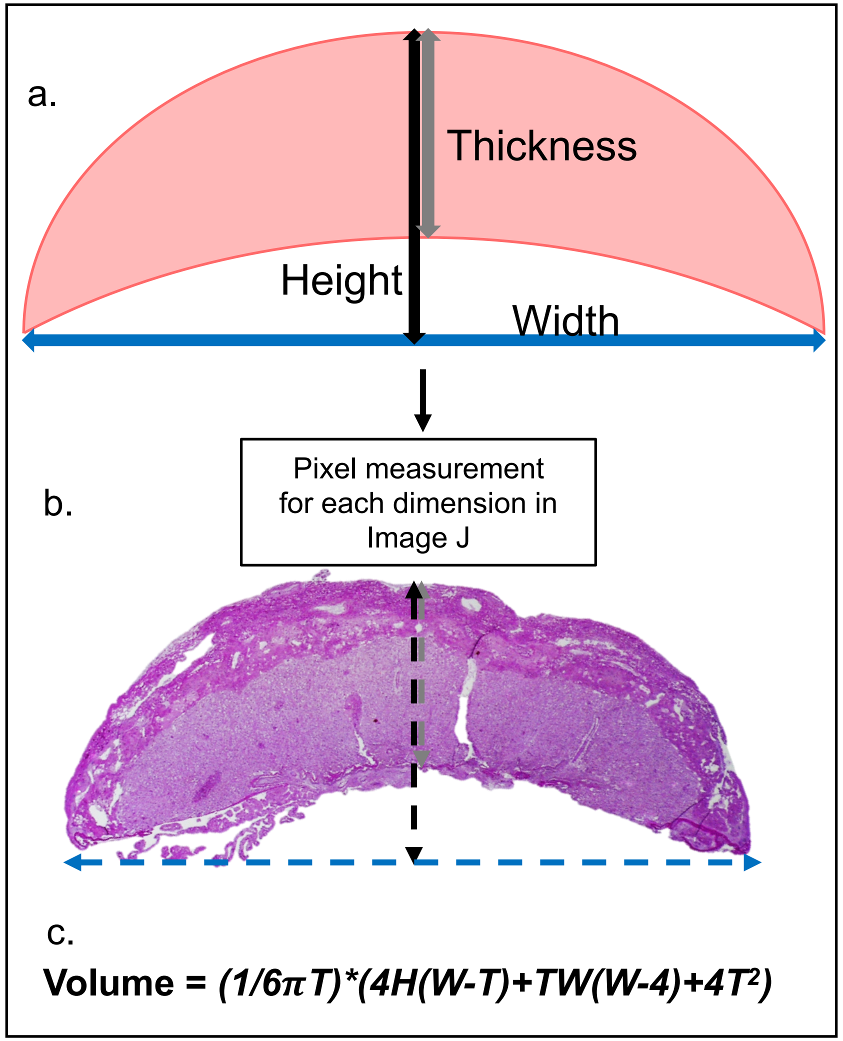

Fig. S3. Method used to measure placenta height, thickness, width and volume. a. Placenta diagram showing dimensions (solid arrows indicate thickness, height or width) measured using the image J software. b. Representative placenta image with dashed arrows depicting different dimensions. c. Formula used to calculate placenta volume from measurements of height (H), width (W), and thickness (T) adapted from Azpurua et al, 2009.

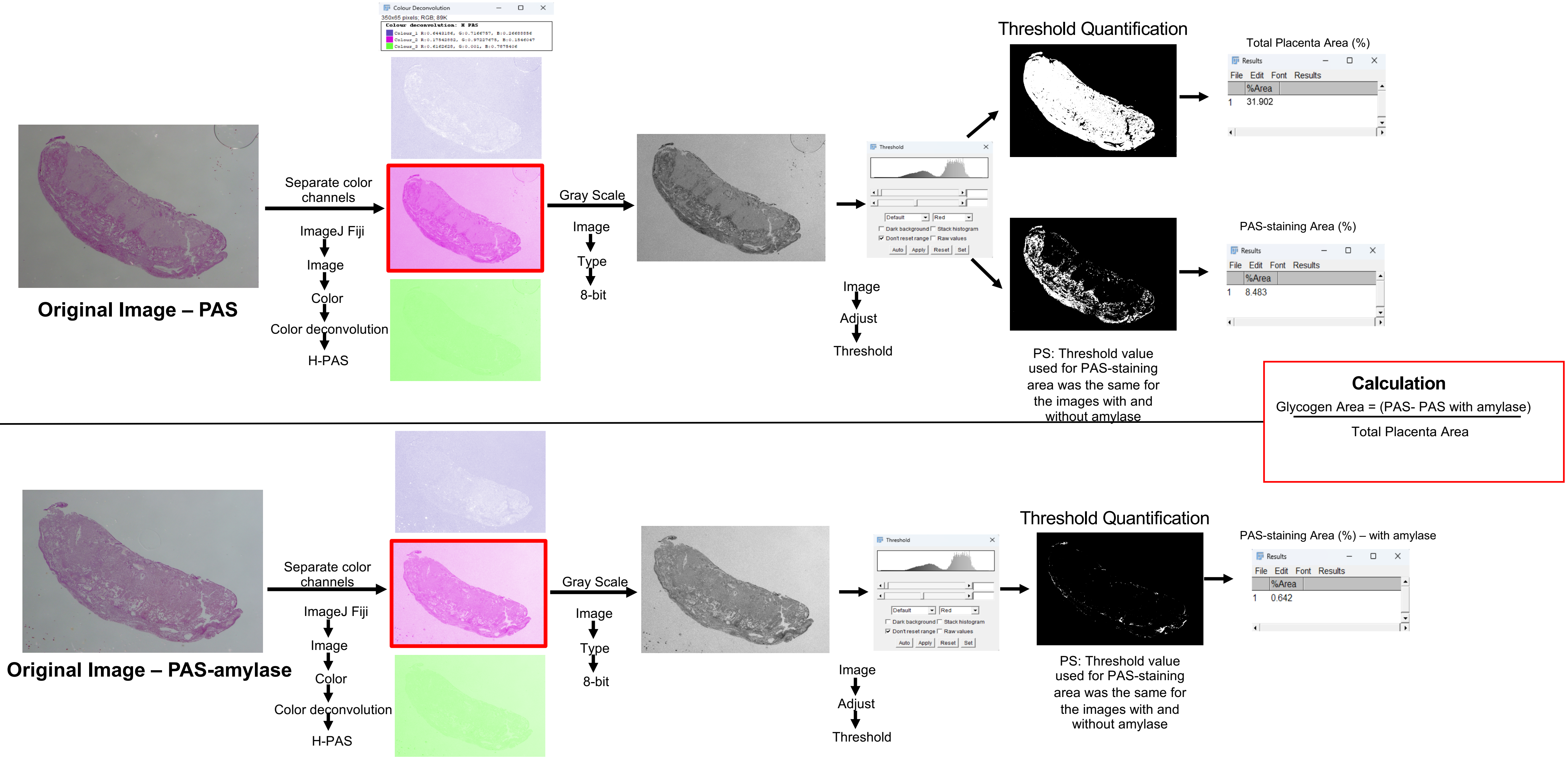

Fig. S4. Method to quantify placenta glycogen levels: Placenta glycogen content was measured via periodic acid–Schiff (PAS) staining, with or without with α-amylase digestion. To quantify placenta glycogen levels, two serial PAS-stained placenta sections (with and without alpha-amylase treatment) were analyzed using the ImageJ Fiji image processing software. Using the H PAS function within ImageJ Fiji, color filters were separated, and then the intensity of PAS staining (pixels) was quantified for both section images. Glycogen levels were measured using the difference in color intensity between images without and with amylase and normalized by total placenta area.

Fig3d- Membrane 1

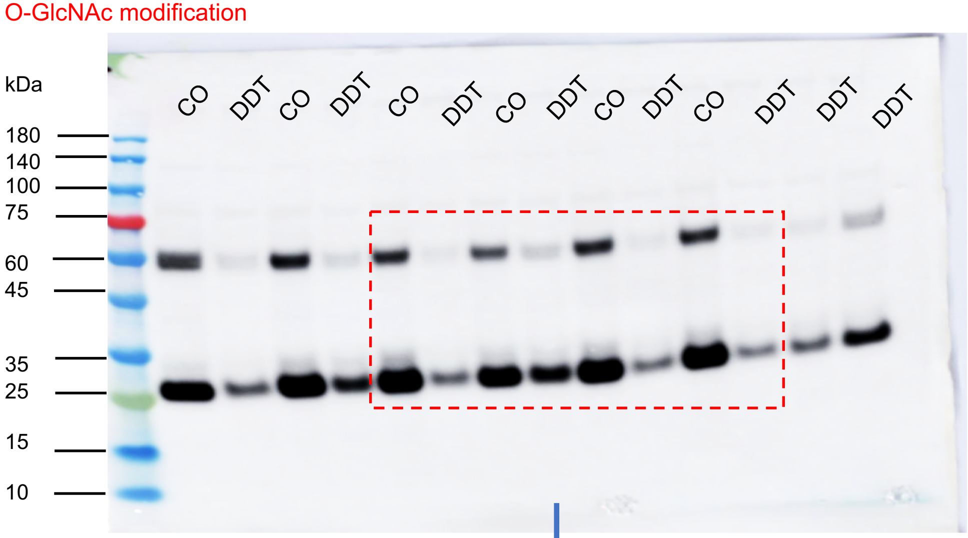

Original membrane was incubated with antibody against O-GlcNAc modification of protein in placenta lysates.

The membrane was then cut into two, stripped and incubated with antibodies against OGT (~110Kda) or Beta-actin (42 Kda) as shown below

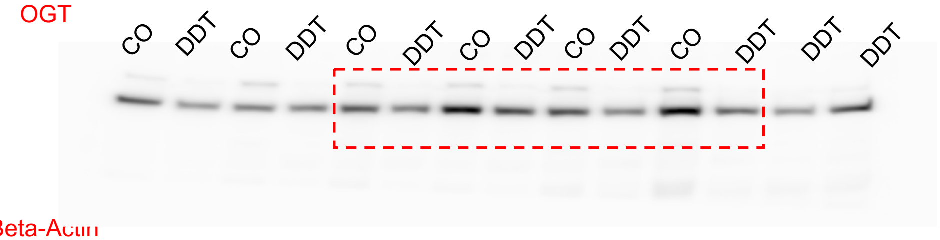

1. The top half of membrane was incubated with antibody against OGT (~110kDa).

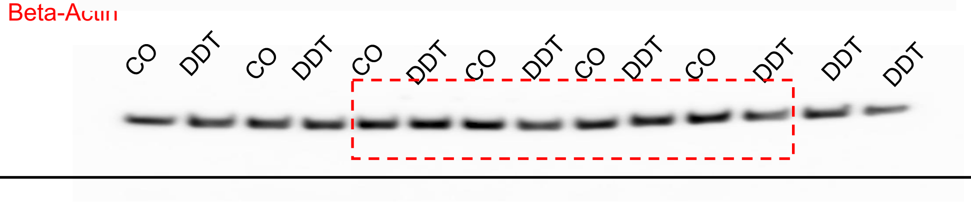

2. The bottom half of membrane was incubated with Beta-actin antibody (42 kDa)

Fig3g- Membrane 2

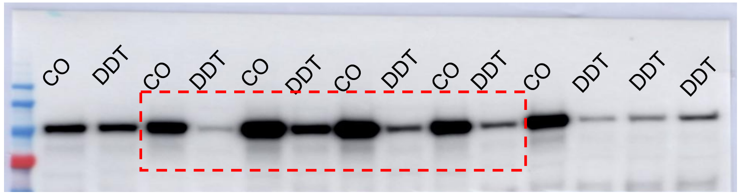

1. The top half of membrane was incubated with antibody against EZH2 (~95 kDa).

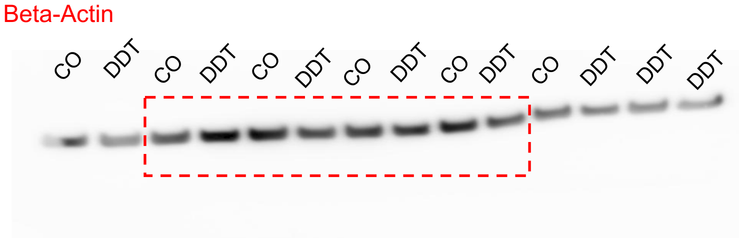

2. The bottom half of membrane was incubated with Beta-actin antibody(42 kDa).

Fig3i- Membrane 3

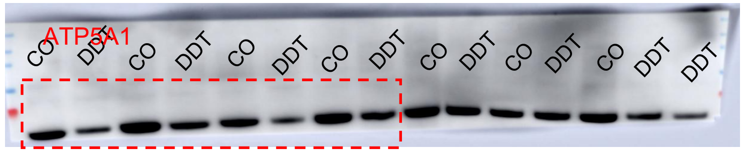

1. The top half of membrane was incubated with antibody against ATP synthase (~60 kDa).

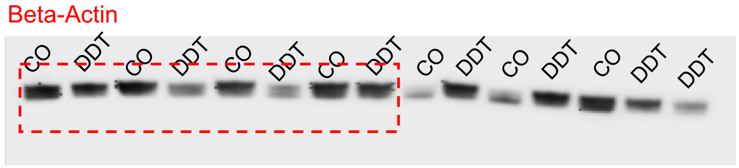

2. The bottom half of membrane was incubated with Beta-actin antibody (42 kDa).

Fig. S5. Uncropped Western Blot membranes shown in Figure 3- **Male placentas lysates** of DDT or CO offspring. Data quantification is shown in Figure 3e-f and Figure 3 i-j

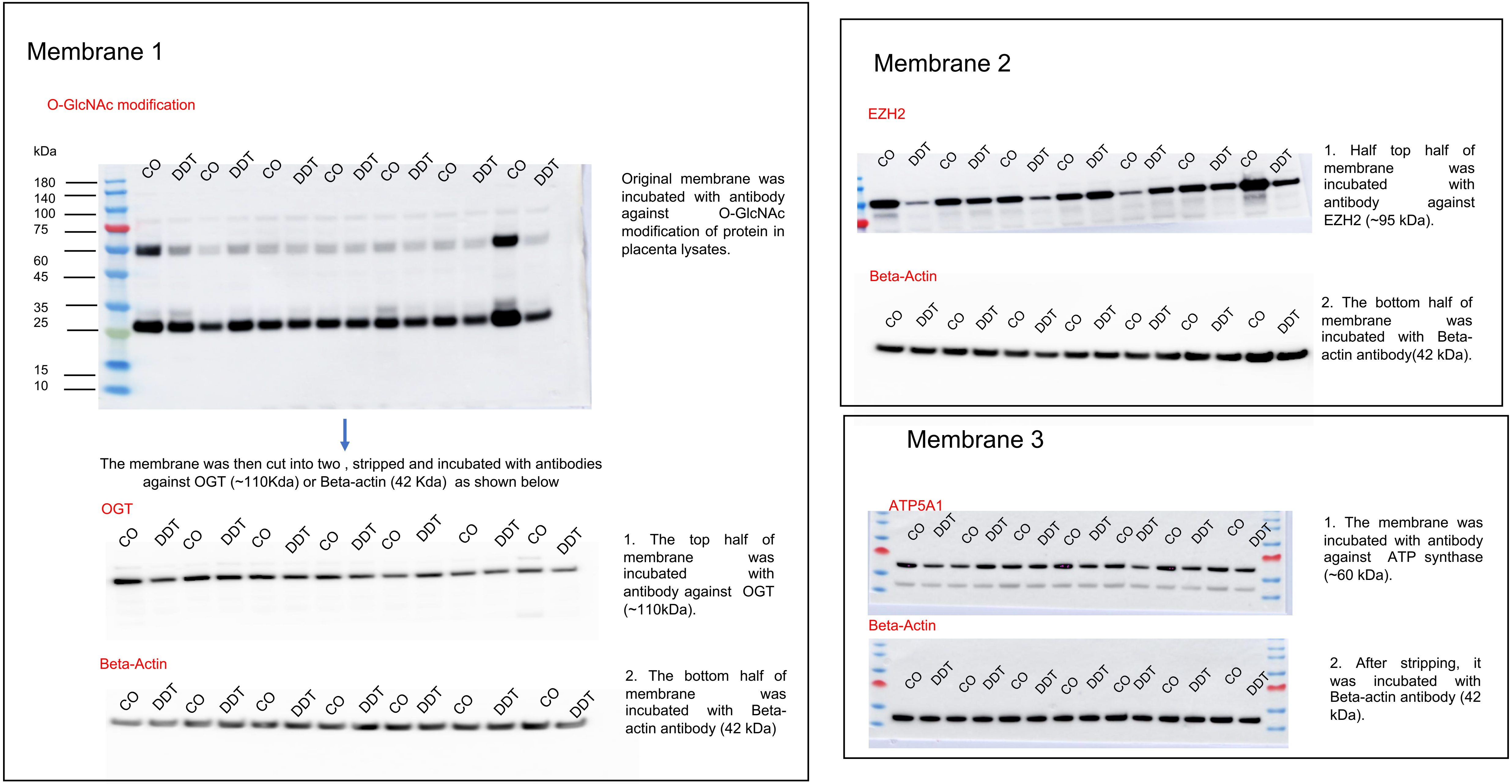

Fig. S6. Uncropped Western Blot membranes in **female placentas lysates** of DDT or CO offspring (not shown in Figure 3). Data quantification is shown in Figure 3e-f and Figure 3 i-j

Fig.7h-i. Membrane

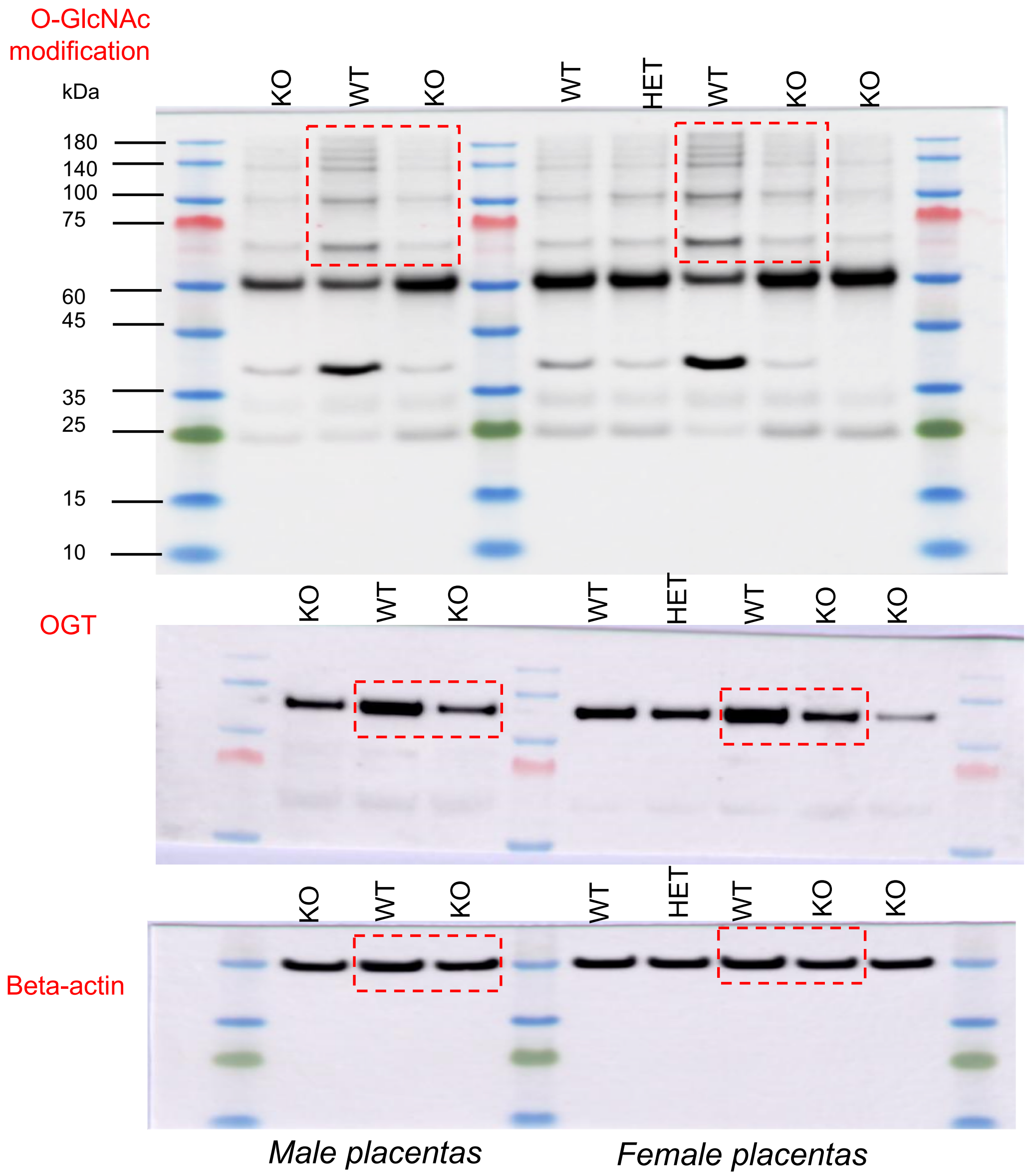

Original membrane was incubated with antibody against O-GlcNAc modification of protein in placenta lysates.

1. After stripping, top half of membrane was incubated with antibody against OGT (~95-100kDa).

2. After stripping, the bottom half of membrane was incubated with Beta-actin antibody (42 kDa)

Fig. S7. Uncropped Western Blot membranes shown in Figure 7- Male and female OGT WT or KO placentas
